# Biomonitoring of Urinary Benzene Metabolite SPMA in the General Population in Central Italy

**DOI:** 10.3390/toxics6030037

**Published:** 2018-07-11

**Authors:** Giovanna Tranfo, Daniela Pigini, Enrico Paci, Lisa Bauleo, Francesco Forastiere, Carla Ancona

**Affiliations:** 1 INAIL Research, Department of Occupational and Environmental Medicine, Epidemiology and Hygiene, Via di Fontana Candida 1, 00078 Monte Porzio Catone, Italy; g.tranfo@inail.it (G.T.); d.pigini@inail.it (D.P.); e.paci@inail.it (E.P.); 2 Lazio Regional Health Service, Department of Epidemiology, Via Cristoforo Colombo, 112, 00147 Rome, Italy; fran.forastiere@gmail.com (F.F.); c.ancona@deplazio.it (C.A.)

**Keywords:** human biomonitoring, urine, non-occupational exposure, S-phenyl-mercapturic acid, HPLC-MS/MS

## Abstract

Background: Benzene is an important component of cigarette smoke and car exhaust. Products containing benzene in concentrations greater than 0.1% are prohibited in Europe, but 1% of benzene is still allowed in gasoline. The purpose of the study was to assess the levels of urine benzene biomarkers in a sample of the general population not occupationally exposed to benzene, resident in the period 2013–2014 in Central Italy, compared to other groups. Methods: The urinary levels of the benzene metabolites S-phenyl-mercapturic acid (SPMA) and cotinine (nicotine metabolite) were determined by means of HPLC with mass spectrometric detection in 1076 subjects. Results: The median SPMA value in smokers was 1.132 µg/g of creatinine while in non-smokers it was 0.097 µg/g of creatinine, and the 95th percentile results were seven times higher. Conclusion: The main source of benzene exposure in the studied population was active smoking, however, non-smokers were also exposed to airborne benzene concentrations. The concentration ranges found in this study can be used as a background reference for occupational exposure assessment to benzene by means of SPMA biomonitoring.

## 1. Introduction

Benzene is an important chemical compound used in the manufacturing of polymers, plastics, rubber, dyes, detergents, and other products, and it is a ubiquitous pollutant of indoor and outdoor air. In fact, even though in Europe the sale to or use by consumers of products that contain benzene in concentrations greater than 0.1% by weight is restricted by the European regulation on Registration and Evaluation of Chemicals (REACH), human exposure to this substance can still be due to industrial use, combustions of organics and natural gas, and motor fuels, as 1% in volume of benzene is allowed in gasoline according to Directive 98/70/EC [1].

The Directive 2008/50/EC on ambient air quality and cleaner air for Europe sets objectives for ambient air quality in order to protect human health and the environment as a whole; these objectives relate to sulphur dioxide, nitrogen dioxide, particulate matter, lead, benzene, and carbon monoxide. For benzene, the limit value has been set at 5 μg/m^3^ as the annual average since 1 January 2010 [2].

Benzene exposure for industrial sectors can be easily assessed if the quantity of material used and the working environment are well defined, while for the general population, it is harder to quantify because individual lifestyles are extremely variable, ambient weather conditions can impact exposure, and living environments are different [3].

The air concentrations for different employment sectors can range from 1 µg/m^3^ to over 1000 µg/m^3^ (aviation workers, service station workers, bus drivers, police, urban workers, fishermen, and shoe production workers), but in the last decades, in developed countries, airborne benzene has progressively decreased as a consequence of preventive actions [4]; furthermore, outdoor ambient air concentrations of benzene are dependent on geographical location (i.e., rural versus urban). Benzene is also an important component of cigarette smoke. The contribution of environmental tobacco smoke is a major cause of indoor benzene exposure and depends upon local restrictions on smoking. Other significant sources of indoor benzene exposure are incense burning and traffic emissions. 

Benzene is a known carcinogen and causes hematotoxicity at exposure levels below 1 ppm (3.25 mg/m^3^) [5], which is the occupational limit value in the Recommendation from the Scientific Committee on Occupational Exposure Limits for benzene [6]. Exposure to a time-weighted average (TWA) of 1–5 parts per million (ppm) benzene in ambient air for 40 years is associated with an increased risk of acute myeloid leukemia [7]. The capacity of individuals to metabolize benzene is modulated by genetic factors [8]. Benzene uptake occurs mainly by inhalation in both occupational and non-occupational exposure. Benzene is oxidized to benzene oxide (BO) from cytochrome P450 (CYP) oxidase. A small fraction of BO (about 1% of total benzene uptake at 0.1–10 ppm of exposure) is detoxified through conjugation with glutathione (GSH) by glutathione-S-transferases (GSTs), and excreted in urine as S-phenyl-mercapturic acid (SPMA) [9]. S-phenyl-mercapturic acid (SPMA) is a very specific urinary biomarker of benzene. Its half-life is in a range of 9–13 h, and its accumulation is not probable, therefore, it is a biomarker of recent exposure [3].

Human biomonitoring data integrates all sources of possible exposure to a chemical, but it does not provide information on a single route of exposure. Biomonitoring permits an assessment of the exposure by quantitating a dose biomarker in biological fluids. The knowledge of the concentration of a chemical substances in biological fluids (biomarkers) measured in subjects without occupational exposure, accounts for the biological variability of the examined population and of other factors like residence and lifestyle [3,10]; it is also important for the evaluation of action levels or biological exposure limit (both environmental and occupational). 

Cigarette smoking is a major source of exposure to benzene in active smokers [4] and is also able to affect the levels of biological markers of exposure to benzene in non-smokers exposed to passive smoking [11,12].

It has been estimated that smokers receive about 90% of their benzene intake from smoking [7].

An Italian surveillance study showed that during the period from 2014–2017 in the population of 18 and 69-years-olds, non-smokers were the majority (56.4%), ex-smokers (17.6%) were the minority, and 26% of the population were active smokers [13].

Exposure to benzene of the general population has been reduced significantly in Europe both in the outdoors and indoors by lowering the benzene content in gasoline and prohibiting smoking in many public places; therefore, population biomarker values are changing over time, and must be periodically updated.

The objective of the present study was to provide a measure of the environmental benzene exposure, according to smoking status and occupation, during the period from 2013–2014 in a sample of the general population living in Central Italy with no declared occupational exposure to benzene. 

## 2. Materials and Methods

### 2.1. Study Population

The present study is part of a larger biomonitoring study, in which samples were collected between May 2013 and December 2014 from a population randomly selected from the municipality registers of the area of Civitavecchia (Italy) from the about 130,000 inhabitants [14]. The study protocol was approved by the local ethics committee. The subjects who agreed to participate to the study gave a written informed consent and filled in a questionnaire for collecting information on age, lifestyle, and food habits, cigarette, cigar, or tobacco smoking, the starting age for smoking, the end age for smoking for ex-smokers, electronic cigarette smoking, passive smoking, drug use, working activities, hobbies, use of chemical products, and in particular the possible occupational exposure to benzene. Part of this information was collected for the purpose of studying exposure to different chemical pollutants and was not used in the present study. The group studied included 1076 subjects aged 35–69 years. Information about the occupation status (employed, nor employed, housewives, retired) of participants was assessed during the interview. Housewife was considered a separate occupation. The smoking status was assessed using the urinary concentration of cotinine, and the cutoff value for the definition of smoker was set at urinary cotinine ≥100 µg/g of creatinine [15].

### 2.2. Urine Sample Collection and Preparation

Fasting subjects collected the first urine of the morning in empty plastic sterile containers on the same day as the medical visit: 30 mL of each sample were transferred into 50 mL Teflon tubes, identified with the subject code, frozen at −20 °C, and later transported to the laboratory where they were stored at −20 °C until analysis. Urinary creatinine was determined by the method of Jaffè using alkaline picrate test with UV/Vis detection at 490 nm [16]. The samples having a creatinine concentration higher than 3 g/L or lower than 0.3 g/L were discarded and the corresponding volunteers were excluded from the study in accordance with the American Conference of Governmental Industrial Hygienists (ACGIH) recommendation (ACGIH, Cincinnati 2014) [17].

### 2.3. Analytical Method

The concentration of the benzene and nicotine metabolites, SPMA and cotinine, was determined by isotopic dilution HPLC-MS/MS according to an analytical method previously validated in our laboratories [15]. Briefly, 3 mL of urine were acidified at pH 2, in order to hydrolyze the precursor of SPMA, with the deuterium labeled internal standards. Solid phase extraction (SPE) purification was carried out on Sep-pack C18 cartridges in two steps in order to elute a first fraction containing the acidic metabolite (SPMA) and subsequently a second one at pH 8 containing the cotinine: the two fractions were then injected separately into the API 4000 HPLC-MS/MS system. The HPLC-MS/MS is a Series 200 LC quaternary pump (PerkinElmer, Norwalk, CT, USA) coupled with an AB/Sciex API 4000 triple-quadrupole mass spectrometry detector equipped with a Turbo Ion Spray (TIS) probe. A Sinergi Fusion C18 analytical column (150 × 4.6-mm, 4-μm) was used for the analysis of urine samples and for the calibration standards for SPMA and cotinine. The mobile phase was a linear gradient of acetonitrile and acetic acid 1.0% *v*/*v* in water, flow rate 600 μL/min. The total run time was 10 min for the SPMA and 5 min for the cotinine. The precursor→product ionic transitions monitored were 238.1→109.1 for SPMA and 240.1→109.1 for SPMAd_2_ (in the negative ion mode) and 177.3→80.10 for cotinine and 180.3→80.10 for cotinine-d_3_ (in the positive ion mode).

The precision for SPMA at the calibration level of 2 μg/L is 3%, and at the lowest level it is 10%. The limits of detection (LOD) calculated using the approach based on the standard deviation of the response and the slope, and expressed as 3.3 σ/S, were 0.026 µg/L for SPMA and 12.41 µg/L for cotinine. The final concentrations of both analytes were expressed in µg/g of creatinine to normalize values with respect to urine dilution variability. SPMA data below the LOD accounted for 20% of the population, and they have been substituted with the value of ½ LOD in order to calculate the geometric mean (GM) and perform other statistical analysis.

### 2.4. Statistical Analysis

Descriptive statistics were carried out, and the SPMA concentration is presented as geometric mean (GM) with its geometric standard deviation (GSD), 5th, 50th, and 95th percentile. The relation among SPMA urinary concentrations and demographic characteristics (gender, age, occupation) in non-smokers was assessed using a linear regression model in which the dependent variable was the log-transformed SPMA. In this case, the measure of risks is expressed in term of Geometric mean ratio (GMR). Confidence interval of 95% were calculated. SAS (SAS Institute Inc., Cary, NC, USA) and STATA version 13 (StataCorp, College Station, TX, USA) software programs were used for the statistical analyses.

## 3. Results

Table 1 describes the characteristic of the population sample by smoking status.

Active smokers were 27.5% of the sample, females 57.2% of the sample, and 53.5% were employed. In the study sample, active smokers were younger, and there were no subjects with declared occupational exposure to benzene as assessed from the ABC study interview. 

Table 2 reports the distribution of SPMA concentrations expressed in µg/g creatinine (Geometric mean and <25th, 25th–50th, 50th–75th, >75th percentile cut-offs) by the main characteristics of the ABC sample according to smoking status, assessed by means of the cotinine concentration of the same urine samples. Values expressed in µg/L are reported in the Supplemental Material (Table S1). Reference values for the biological monitoring of occupational exposures are generally normalized on the basis of creatinine concentration or specific gravity to account for fluctuations in urine dilution. We preferred the use of creatinine as there are many more published results for comparison, and because this is the parameter used by the ACGIH.

Among smokers, we found higher SPMA concentrations (µg/g creatinine) in females (GSD (SD) 1.120 (4.612)) and people aged 55–64 years (1.262 (4.392)); housewives showed the highest values ((1.533 (4.827) when compared with the other occupational categories. In non-smokers, the median SPMA concentration was 0.1 μg/g of creatinine, with the 95th percentile equal to 0.7 μg/g of creatinine. No particular differences in gender, age, and occupation categories were observed, although the highest value was found in the oldest /retired subjects (aged ≥ 55), while retired and unemployed showed the highest median. 

Table 3 shows results from the multivariate approach; when adjusting for participants’ gender, age, and occupation, we found that SPMA concentrations were higher in females compared to males (*p* = 0.053) while no differences were found between age groups and occupational categories.

## 4. Discussion

Human biomonitoring is a powerful tool used in national and international surveys to assess the integrated exposure of the population to xenobiotics from different sources. In the population studied, the main source of exposure to benzene was active smoking: the data show that the mean SPMA value in smokers is about ten times that of non-smokers. The highest SPMA level was found in female smokers: women present a higher concentration of metabolites both when normalized for the creatinine and when not (µg/L), even when considering that creatinine concentrations are usually lower than in men.

The assessment of benzene exposure by means of biological monitoring does not permit source apportionment as it is integrated information. In the study sample, there were no subjects with declared occupational exposure to benzene and, therefore, benzene metabolites found in non-smokers could derive from passive smoking, use of incense in the home, or exposure to traffic. The median SPMA concentration of non-smokers was 0.1 μg/g of creatinine, that refers only to 50% of the sample, while the 95th percentile was 0.7 μg/g of creatinine, that is seven times higher than the median. This means that efforts to avoid or reduce benzene exposure should be made at the individual level by subjects having the higher biomarker (exposure) values to reach the lower ones.

Non-smokers’ values were further stratified by age and occupational classes to be available as reference or “controls” for studies on subjects with similar characteristics. The highest value was found in the oldest/retired subjects (aged ≥ 65), while unemployed show the highest median. A possible explanation for these differences can be found in life habits (all these subjects have more free time) or in metabolic parameters (slower metabolism, more fat tissue). The same exercise cannot be done for the smokers, as any difference would mainly be due to the number of cigarettes.

We also examined other published data regarding urinary concentrations of SPMA in subjects without occupational exposure, all analytically determined by means of HPLC-MS/MS and published from 2011 to 2015. Only seven papers reported such values, four of which referred to population studies [18,19,20,21] and three to controls in studies on occupational exposures [22,23,24].

Comparison of our results with data from the literature shows that other studies included significantly fewer subjects than this one. Mean and median SPMA urinary concentrations are comparable to those found in the five studies on subjects who were resident in Italy, while the geometric mean found in this study for non-smokers is lower than those reported for African [20] and Chinese subjects [19], where airborne benzene concentrations are apparently higher. Moreover, none of the recent studies explored different age or job classes. Results summarized in Tables are reported as Supplemental Material (Tables S2 and S3).

## 5. Conclusions

Tobacco smoke (active or passive) is confirmed to be the main source of benzene exposure for the general population, and it is the most important confounding factor in the biological monitoring of occupational and environmental exposure to benzene, especially when exposure levels are low and very low. However, benzene exposure can still be high in non-smokers, and individual exposure to benzene can be reduced.

Biological values determined in the general population can be used to understand whether the levels found in workers are indicative of a professional exposure. This is particularly important for substances for which health-based exposure-limit values have not been defined, and it is particularly useful to aid in the interpretation of biological monitoring for genotoxic carcinogens. The comparison should be made taking into account the possible confounding factors, like smoking, that can produce significantly higher levels of urinary SPMA than does the occupational exposure to benzene. The values presented for urinary SPMA in this paper are a sound basis for the definition of occupational exposure to benzene in non-smoking subjects who reside and work in Italy. For smokers, due to the low benzene exposure values reached because of the European legislation on occupational and environmental safety and health, the amount of SPMA produced by smoking could completely mask the airborne benzene contribution. However, SPMA levels higher than the 95th percentile of the smokers group found in this paper should be further investigated while also considering the cotinine concentration of the subjects.

It must be stressed that environmental levels of airborne benzene should further decrease in time and, therefore, biological values, especially for non-smokers, should be reassessed periodically.

## Figures and Tables

**Table 1:** Characteristics of the study population by smoking status.

Population Group		Total		Smokers		Non Smokers	
		1076		296		780	
**Gender**	Males	461	42.8%	117	39.5%	344	44.1%
	Females	615	57.2%	179	60.5%	436	55.9%
**Age group (years)**	35–44	233	21.7%	78	26.4%	155	19.9%
	45–54	335	31.1%	93	31.4%	242	31.0%
	55–64	323	30.0%	90	30.4%	233	29.9%
	35–69	185	17.2%	35	11.8%	150	19.2%
**Occupation**	Employed	576	53.5%	173	58.4%	403	51.7%
	Unemployed	53	4.9%	19	6.4%	34	4.4%
	Housewives	217	20.2%	58	19.6%	159	20.4%
	Retired/Disabled	229	21.3%	46	15.5%	184	23.6%

**Table 2:** Urinary S-phenyl-mercapturic acid (SPMA) concentrations (µg/g creatinine).

Group		N.	Geometric Mean (GSD)	5th Percentile	50th Percentile	95th Percentile	Min–Max
All subjects		1076	0.139 (7.409)	<LOD	0.151	3.403	<LOD–15.487
**Smokers (cotinine ≥ 100 µg/g creatinine)**							
**All**		296	0.926 (4.708)	0.058	1.132	6.612	<LOD–15.487
**Gender**	Males	117	0.691 (4.669)	0.053	0.710	6.02	<LOD–15.487
	Females	179	1.120 (4.612)	0.078	1.505	7.464	<LOD–11.917
**Age group (years)**	35–44	78	0.800 (4.699)	0.052	1.101	5.739	<LOD–10.972
	45–54	93	0.806 (4.729)	0.069	0.967	6.088	<LOD–9.887
	55–64	90	1.262 (4.392)	0.107	1.701	9.150	<LOD–15.487
	35–69	35	0.841 (5.283)	0.081	1.259	6.166	<LOD–11.917
**Occupation**	Employed	173	0.767 (4.628)	0.06	0.940	5.981	<LOD–15.487
	Unemployed	19	0.846 (7.116)	<LOD	0.979	7.180	<LOD–7.948
	Housewives	58	1.533 (4.827)	0.095	2.447	8.355	<LOD–11.917
	Retired	46	0.785 (4.245)	0.102	1.103	3.858	<LOD–5.702
**Non Smokers (cotinine < 100 µg/g creatinine)**							
**All**		780	0.068 (5.236)	<LOD	0.097	0.699	<LOD–2.667
**Gender**	Males	344	0.059 (5.522)	<LOD	0.088	0.633	<LOD–2.667
	Females	436	0.075 (4.985)	<LOD	0.103	0.706	<LOD–2.380
**Age group (years)**	35–44	155	0.059 (5.475)	<LOD	0.079	0.597	<LOD–1.480
	45–54	242	0.058 (5.479)	<LOD	0.089	0.562	<LOD–1.283
	55–64	233	0.081 (4.668)	<LOD	0.115	0.671	<LOD–2.380
	35–69	150	0.075 (5.400)	<LOD	0.098	1.000	<LOD–2.667
**Occupation**	Employed	403	0.061 (5.399)	<LOD	0.094	0.658	<LOD–1.283
	Unemployed	34	0.080 (5.224)	<LOD	0.117	0.710	<LOD–1.480
	Housewives	159	0.077 (4.471)	<LOD	0.099	0.625	<LOD–1.463
	Retired	184	0.080 (5.149)	<LOD	0.105	0.907	<LOD–2.667

GSD: geometric standard deviation; LOD: Limit of detection, for SPMA 0.026 μg/L.

**Table 3:** Association between urinary concentration of SPMA and gender, age, and occupation. GMR = geometric mean ratio.

Group		GMR	C.I. 95%			*p*-Value
**Gender**	Males	1.00				
	Females	1.28	1.00	-	1.65	0.053
**Age group (years)**	35–44	1.00				
	45–54	1.03	0.75	-	1.41	0.848
	55–64	1.29	0.92	-	1.80	0.144
	>65	1.03	0.66	-	1.61	0.898
**Occupation**	Employed	1.00				
	Unemployed	1.15	0.66	-	1.98	0.622
	Housewives	0.95	0.69	-	1.32	0.782
	Retired	1.27	0.88	-	1.85	0.208

## Supplementary Materials

The following are available online at http://www.mdpi.com/2305-6304/6/3/37/s1, Table S1. Urinary SPMA concentrations (µg/L); Table S2. Summary of the population studies published from year 2011; Table S3. Summary of the occupational exposure studies published from year 2011 (data of controls).
