# Supplementary material for: Biomonitoring of Urinary Benzene Metabolite SPMA in the General Population in Central Italy
Source: Toxics. 2018 Jul 11;6(3):37. doi: 10.3390/toxics6030037 (PMC6161128; doi:10.3390/toxics6030037)
Supplement: Supplementary file 1 [file toxics-06-00037-s001.pdf]

Supplementary Materials: Biomonitoring of Urinary Benzene Metabolite SPMA in the General Population in Central Italy

Giovanna Tranfo, Daniela Pigini, Enrico Paci, Lisa Bauleo, Francesco Forastiere and Carla Ancona

Table S1. Urinary SPMA concentrations (μg/L).

Group		N.	Geometric mean (GSD)	5th *percentile*	50th *percentile*	95th *percentile*	Min-Max
**All subjects**		**1076**	0.144 (7.861)	< LOD	0.170	3.626	< LOD–14.450
**Smokers**							
**All**		**296**	1.072 (4.699)	0.066	1.498	6.401	< LOD–14.450
**Gender**	Males	**117**	0.691 (4.669)	0.053	0.710	6.02	<LOD–15.487
	Females	**179**	1.120 (4.612)	0.078	1.505	7.464	<LOD–11.917
**Age group (years)**	35-44	**78**	0.800 (4.699)	0.052	1.101	5.739	< LOD–10.972
	45-54	**93**	0.806 (4.729)	0.069	0.967	6.088	< LOD–9.887
	55-64	**90**	1.262 (4.392)	0.107	1.701	9.150	< LOD–15.487
	>65	**35**	0.841 (5.283)	0.081	1.259	6.166	< LOD -11.917
**Occupation**	Employed	**173**	0.767 (4.628)	0.06	0.940	5.981	<LOD–15.487
	Unemployed	**19**	0.846(7.116)	< LOD	0.979	7.180	< LOD–7.948
	Housewives	**58**	1.533 (4.827)	0.095	2.447	8.355	< LOD–11.917
	Retired	**46**	0.785 (4.245)	0.102	1.103	3.858	< LOD–5.702
**Non Smokers**							
**All**		**780**	0.068 (5.236)	< LOD	0.097	0.699	< LOD–2.667
**Gender**	Males	**344**	0.059(5.522)	<LOD	0.088	0.633	<LOD–2.667
	Females	**436**	0.075 (4.985)	<LOD	0.103	0.706	<LOD–2.380
**Age group (years)**	35-44	**155**	0.059 (5.475)	<LOD	0.079	0.597	< LOD–1.480
	45-54	**242**	0.058 (5.479)	< LOD	0.089	0.562	< LOD–1.283
	55-64	**233**	0.081 (4.668)	< LOD	0.115	0.671	< LOD–2.380
	>65	**150**	0.075 (5.400)	< LOD	0.098	1.000	< LOD–2.667
**Occupation**	Employed	**403**	0.061 (5.399)	< LOD	0.094	0.658	< LOD–1.283
	Unemployed	**34**	0.080 (5.224)	< LOD	0.117	0.71	< LOD–1.480
	Housewives	**159**	0.077 (4.471)	< LOD	0.099	0.625	< LOD–1.463
	Retired	**184**	0.080 (5.149)	< LOD	0.105	0.907	< LOD–2.667

Table S2. Summary of the population studies published from the year 2011.

Subjects	SPMA	Analytical technique	Country	Reference
395 Children	Mean (SD) 0.62 (0.56); 5-95th perc. 0.11–1.83; GM 0.44; Range (0.06–4.35)	HPLC-MS/MS μg/g creatinine	Italy	[1]

Women non smoking 99 aged 45-54	GM 0.12; Range (0.10–0.14)	HPLC-MS/MS µg/g creatinine	China	[2]
125 aged 55-64	GM 0.12; Range (0.10–0.13)			
104 aged 65-74	GM 0.13; Range (0.12–0.15)			
All: 220	Mean 1.3; Median 1.0; 95th perc. 8.1; GM 1.0; Range (0.1–9.6)	HPLC–MS/MS µg/L	Africa	[3]
141 Non-smokers	Mean 0.6; Median 0.5; 95th perc. 7.2; GM 0.3; Range (0.1–8.2)			
79 Smokers	Mean 2.1; Median 1.6; 95th perc. 9.0; GM 1.7; Range (0.8–9.6)			
111 Females	Mean 1.4; Median 1.3; 95th perc. 8.2; GM 1.2; Range (0.1–9.6)			
109 Males	Mean 1.1; Median 0.9; 95th perc. 8.0; GM 0.9; Range (0.1–9.0)			
50 aged 6-14	Mean 0.8; Median 0.6; 95th perc. 7.0; GM 0.4; Range (0.1–8.2)			
170 aged >14	Mean 1.9; Median 1.5; 95th perc. 9.3; GM 1.6; Range (0.1–9.6)			
All: 103	Mean 0.54; Median <0.10 5 - 95th perc. <0.10–3.83	HPLC-MS/MS µg/L	Italy	[4]

GM: geometric mean

Table S3. Summary of the occupational exposure studies published from the year 2011 (data of controls).

Controls	SPMA	Analytical technique	Country	Reference
All: 108<br>Non-smokers: 65<br>Smokers: 43	Median <0.10; 5-95th perc. <0.10–1.79<br>Median <0.10; 5t-95th perc. <0.10–0.18<br>Median 0.24; 5- 95th perc. <0.10–1.89	HPLC–MS/MS µg/g creatinine	Italy	[5]
All: 31	Mean (SD) 0.65 (1.00)<br>Median 0.22; Range (0.03–4.48)	HPLC-MS/MS µg/g creatinine	Italy	[6]
All: 65<br>Non-smokers: 51<br>Smokers: 14	Median 0.2; Range (<0.1–0.3)<br>Median 0.1; Range (<0.1–0.2)<br>Median 0.5; Range (<0.2–1.5)	HPLC/MS µg/L	Italy	[7]

References

1. Protano, C.; Andreoli, R.; Manini, P.; Vitali, M. Urinary trans, trans-muconic acid and S-phenyl-mercapturic acid are indicative of exposure to urban benzene pollution during childhood. *Sci. Total Environ.* **2012**, *435–436*, 115–123, doi:10.1016/j.scitotenv.2012.07.004.
2. Hecht, S.S.; Koh, W.-P.; Wang, R.; Chen, M.; Carmella, S.G.; Murphy, S.E.; Yuan. J.-M. Elevated Levels of Mercapturic Acids of Acrolein and Crotonaldehyde in the Urine of Chinese Women in Singapore Who Regularly Cook at Home. *PLoS ONE* **2015**, *10*, e0120023, doi:10.1371/journal.pone.0120023.
3. Tuakuila, J. S-phenyl-mercapturic acid (S-PMA) levels in urine as an indicator of exposure to benzene in the Kinshasa population. *Int. J. Hyg. Environ. Health* **2013**, *216*, 494–498, doi:10.1016/j.ijheh.2013.03.012.
4. Ranzi, A.; Fustinoni, S.; Erspamer, L.; Campo, L.; Gatti, M.G.; Bechtold, P.; Bonassi, S.; Trenti, T.; Goldoni, C.A.; Bertazzi, P.A.; et al. Biomonitoring of the general population living near a modern solid waste incinerator: A pilot study in Modena, Italy. *Environ. Int.* **2013**, *61*, 88–97, doi:10.1016/j.envint.2013.09.008.
5. Fustinoni, S.; Campo, L.; Mercadante, R.; Consonni, D.; Mielzynska, D.; Bertazzi, P.A. A quantitative approach to evaluate urinary benzene and S-phenyl-mercapturic acid as biomarkers of low benzene exposure. *Biomarkers* **2011**, 1–12, doi:10.3109/1354750x.2011.561499.

6. Lovreglio, P.; Barbieri, A.; Carrieri, M.; Sabatini, L.; Fracasso, M.E.; Doria, D.; Drago, I.; Basso, A.; D'Errico, M.N.; Bartolucci, G.B.; et al. Minore validità del benzene urinario rispetto all'acido S-fenilmercapturico nel rilevare l'esposizione occupazionale ed ambientale a concentrazioni molto basse di benzene. *G Ital Med Lav Erg* **2011**, *33*, 117–124.
7. Campagna, M.; Satta, G.; Campo, L.; Flore, V.; Ibba, A.; Meloni, M.; Tocco, M.G.; Avataneo, G.; Flore, C.; Fustinoni, S.; Cocco. P. Biological monitoring of low-level exposure to benzene. *Med. Lav.* **2012**, *103*, 338–346.

© 2018 by the authors. Submitted for possible open access publication under the terms and conditions of the Creative Commons Attribution (CC BY) license (http://creativecommons.org/licenses/by/4.0/).
